# Two Novel Anoxia-Induced Ethylene Response Factors That Interact with Promoters of Deastringency-Related Genes from Persimmon

**DOI:** 10.1371/journal.pone.0097043

**Published:** 2014-05-07

**Authors:** Ting Min, Fang Fang, Hang Ge, Yan-na Shi, Zheng-rong Luo, Yun-cong Yao, Donald Grierson, Xue-ren Yin, Kun-song Chen

**Affiliations:** 1 Laboratory of Fruit Quality Biology/The State Agriculture Ministry Laboratory of Horticultural Plant Growth, Development and Quality Improvement, Zhejiang University, Zijingang Campus, Hangzhou, PR China; 2 Key Laboratory of Horticultural Plant Biology, Ministry of Education, Huazhong Agricultural University, Wuhan, PR China; 3 Department of Plant Science and Technology, Beijing University of Agriculture, Beijing, PR China; 4 Plant & Crop Sciences Division, School of Biosciences, University of Nottingham, Sutton Bonington Campus, Loughborough, United Kingdom; Institute of Genetics and Developmental Biology, Chinese Academy of Sciences, China

## Abstract

A hypoxic environment is generally undesirable for most plants and stimulates anaerobic metabolism. It is a beneficial treatment, however, for the removal of astringency from persimmon to improve the fruit quality after harvest. High soluble tannins (SCTs) content is one of most important causes of astringency. High CO_2_ (95%) treatment effectively reduced SCTs in both “Mopan” and “Gongcheng-shuishi” persimmon fruit by causing increases in acetaldehyde. Using RNA-seq and realtime PCR, twelve ethylene response factor genes *(DkERF11-22*) were isolated and characterized, to determine those responsive to high CO_2_ treatment. Only two genes, DkERF19 and DkERF22, showed trans-activation effects on the promoters of deastringency-related genes pyruvate decarboxylase genes (*DkPDC2* and *DkPDC3*) and the transcript levels of these genes was enhanced by hypoxia. Moreover, DkERF19 and the previously isolated DkERF9 had additive effects on activating the *DkPDC2* promoter. Taken together, these results provide further evidence that transcriptome changes in the level of *DkERF* mRNAs regulate deastringency-related genes and their role in the mechanism of persimmon fruit deastringency is discussed.

## Introduction

Low oxygen is a common abiotic stress in plant developmental physiology, which is mainly caused by flooding/submerge. On the contrary, however, reduced oxygen concentration is beneficial for some fruit quality traits, eg. Controlled Amosphere (CA) storage with low oxygen level prolongs storage life and maintains fruit quality [1], [2]. Besides CA storage, one interesting additional advantage of low oxygen improvement of fruit quality occurs in persimmon fruit (*Diospyros kaki*), where it removes undesirable astringency. Development of persimmon fruit is accompanied by the accumulation of proanthocyanidins (PAs; also known as condensed tannins, CTs). CTs are colourless polyphenolic compounds important both for the plant and human, however, the soluble part of CTs (SCTs) make an important adverse contribution to fruit taste by causing astringency [3], [4]. Most persimmon fruit are of the astringent type, which are rich in SCTs even at maturity [5], [6]. Among the artificial treatments, high CO_2_ treatment (with reduced oxygen level) and to a lesser extent ethylene treatment, leads to anaerobic fermentation in persimmon fruit, thus triggering acetaldehyde metabolism [7]–[9]. Acetaldehyde plays important roles in polymerization of SCTs, converting them to insoluble condensed tannins (InSCTs) [10], [11].

Due to the importance of deastringency for persimmon fruit quality, we investigated the molecular mechanisms whereby low oxygen drove deastringency by isolation of eight alcohol dehydrogenase genes (*DkADH*) and pyruvate decarboxylase (*DkPDC*) genes from persimmon fruit and showed that increased *DkADH1*, *DkPDC1* and *DkPDC2* mRNA levels were closely correlated with persimmon deastringency. Transient overexpression of *DkPDC2* in persimmon leaves resulted in lower SCTs content [12]. In addition, several hypoxia-responsive *ERF* transcriptional regulator genes were also isolated, including *DkERF4*, *DkERF5*, *DkERF9* and *DkERF10* [12], [13], but only *DkERF9* and *DkERF10* activated the promoters of *DkPDC2* and *DkADH1*, respectively [12]. These results suggested that *ERF* genes are involved in transcriptional regulation of persimmon fruit deastringency, and also expanded the functional changes involving *ERF* genes, which also include texture [14]–[16], carotenoids [17], ethylene [18], senescence [19] and stress response [20]. However, due to the lack of genome information for persimmon fruit, a genome-wide overview of the effects of *ERF* genes on persimmon fruit deastringency was prevented.

In the model plant Arabidopsis, at least four *ERF* genes, including *HRE1*, *HRE2*, *RAP2.2* and *RAP2.12*, were recently characterized as the main plant oxygen-sensing regulators. These *ERF* genes could transcriptionally regulate *ADH* and *PDC*, and lead to hypoxia tolerance [21]–[26]. Compared with arabidopsis *ERF* genes, only two *DkERF* genes (*DkERF9* and *DkERF10*) were characterized as the transcriptional regulator on persimmon deastringency related genes [12]. Thus, additional *ERF* genes related to deastringency might exist in persimmon. Moreover, the four arabidopsis *ERF* genes were all belong to subfamily VII, the regulatory effect of the *ERF* genes from the other subfamilies on hypoxia response or persimmon deastringency need further investigation.

Following our previous studies, a more comprehensive analysis was performed using RNA-seq and twelve novel *ERF* genes were isolated, in addition to the four studied previously. A dual luciferase assay was used to study their regulatory effects on deastringency-related target genes in ‘Mopan’ and ‘Gong cheng-shui shi’ cultivars which were are both astringent types.

## Materials and Methods

### Fruit Materials

Two persimmon cultivars that respond to deastringency treatment, ‘Mopan’ and ‘Gong cheng-shui shi’, were chosen as materials. The batch of ‘Mopan’ persimmon was the same as that used in our previous report [12]. The other batch of astringent persimmon, ‘Gong cheng-shui shi’, was obtained from a commercial orchard at Gongcheng (Guilin, China) in 2012. The fruit were transported to Zhejiang University (Hangzhou, Zhejiang, China) on the second day after harvest. The 180 fruit were divided into two 90 fruit lots. Treated fruit were exposed to 95% CO_2_ and control fruit were placed in air, both in air-tight plastic containers for 1 day. After treatment, the fruit were held in air at 20°C until the end of the experiment. For each sampling point, fruit flesh samples (without skin and core) were taken from three replicates of four fruit each. The samples were frozen in liquid nitrogen and stored at −80°C until further use.

### Fruit Physiology Evaluation

Fruit firmness measurement was carried out in a TA-XT2i texture analyzer (Stable Micro Systems, Godalming, Surrey, UK), fitted with a 5 mm diameter head, 1 mm/s penetration test rate and 1 mm thickness of peel, according to our previous report [13]. 10 fruit per sampling point were measured at two positions 90° to each other at the fruit equator.

The content of soluble condensed tannins (SCTs), the most important index for astringency, were measured using Folin-Ciocalteu reagent according to the method described in our previous report [13].

### RNA Extraction and cDNA Synthesis

Total RNA was prepared according to the method used in our previous report [27]. The trace amount of genomic DNA in total RNA was digested with TURBO DNA free kit (Ambion). First strand cDNA was synthesized from 1.0 µg DNA-free RNA, using iScript cDNA Synthesis Kit (Bio-Rad). For each sampling point, three biological replicates were used for RNA extraction and the subsequent cDNA synthesis.

### Gene Isolation and Sequence Analysis

The novel *ERF* genes induced by anoxia deastringency treatment were isolated based on RNA-Seq. ‘Mopan’ fruit, 0 d (astringency) and 2 d in CO_2_ treatment (deastringency), were chosen and constructed into two different libraries. The RNA extraction and preparatory procedures were as previously reported [28]. The libraries construction and RNA-Seq was performed and sequences assembled and annotated by the Beijing Genome Institute (BGI) (Shenzhen, China). The data were firstly removed the adapter sequences and low quality sequences and de novo assembled into Unigenes using *SOAPdevono* assembly program (version 1.04) [29] and TGICL [30], with the parameters described in previous report [28]. Furthermore, the Unigenes was annotated by BLASTxing to NCBI non-redundant (nr) database with E-value cutoff of 1e^−5^. Meanwhile, RPKM (Reads per kb per million reads) values were used to calculate the UniGene expression level using SOAPaligner software (Version 2.20, http://soap.genomics.org.cn/soapaligner.html) and DFR value, which was calculated based on P value, was used to identify genes expressed differentially between two samples as described as previously reported [28].

Full-length *ERF* genes were amplified with a SMART RACE cDNA Amplification Kit (Clontech). The sequences of primers used for RACE are described in Table S1. Based on the deduced amino acid sequences, a phylogenetic tree of *ERF* genes was generated using ClustalX (v 1.81) and calculated using Figtree (v1.3.1). The deduced amino acid sequences of homologous genes of Arabidopsis were obtained from TAIR (The Arabidopsis Information Resource).

### Oligonucleotide Primers and Real-time PCR

Oligonucleotide primers for real-time PCR analysis were designed with primer3 (v. 0.4.0, http://frodo.wi.mit.edu/cgi-bin/primer3/primer3_www.cgi). The specificity of primers was determined by melting curves and PCR products resequencing. The sequences of oligonucleotide primers are in Table S2.

Real-time PCR was carried out using a CFX96 instrument (Bio-Rad). The PCR mixtures and reactions were according to our previous report, with Ssofast EvaGreen Supermix (Bio-Rad) [13]. The relative abundance of each gene was calibrated by comparison with a sample of day 0 fruit (set as 1). Abundance of cDNA templates was monitored with *DkActin*, a housekeeping gene [31].

### Dual Luciferase Assay

Dual luciferase assay was performed according to our previous report [12], [14]. Two vectors were used as backbones: (1) pGreen II 0029 62-SK vector (SK) was used for harboring the full-length coding sequences of transcription factors, (2) pGreen II 0800-LUC vector (LUC) was used for harboring promoters. The isolated full-length *DkERF9* and *DkERF10*, as well as promoters of *DkADH1* and *DkPDC2*, were constructed into the two vectors, SK and LUC respectively [12]. New *ERF* genes (*DkERF11-22*) and promoters of *DkPDC3* were constructed into SK and LUC vector respectively, using the primers described in Table S3.

All of the constructs were confirmed by sequencing and were then electroporated into *Agrobacterium tumefaciens* GV3101. The transient assay was performed with *Nicotiana benthamiana* leaves. *Agrobacterium* culture mixtures of TFs (1 ml) and promoters (100 µl) were infiltrated into tobacco leaves by needle-less syringes with the infiltration buffer (10 mM MES, 10 mM MgCl_2_, 150 mM acetosyringone, pH 5.6). Tobacco plants were grown in a growth chamber, with light: dark cycles of 16∶8 h. Three days after infiltration, firefly luciferase and renilla luciferase were assayed using the dual luciferase assay reagents (Promega). For each TF-promoter interaction, three independent experiments were performed (at least five replicates in each experiment).

### Yeast One-hybrid Assay

In order to verify the results obtained from the dual luciferase assay, yeast one-hybrid assays were performed using the Matchmaker Gold Yeast One-Hybrid Library Screening System (Clontech, USA). The promoter of *DkADH1*, *DkPDC2*, *DkPDC3* were constructed into pAbAi vector (primers are listed in Table S4). Due to the auto-activation activities of *DkADH1* and *DkPDC3* promoters, only *DkPDC2* promoter was chosen for interaction test. The *DkPDC2*-AbAi and p53-AbAi were linearized and transformed into Y1HGold. The full-lengths of transcript factor *DkERF19* were subcloned into pGADT7 AD vector (AD) (primers are listed in Table S4). Transformed Y1HGold were cultured on SD/−Leu containing 0–200 ng/ml aureobasidin A at 28°C for 3 d to test interaction. pGADT7-Rec (AD-Rec-P53) was co-transformed with the p53-promoter fragment to Y1HGold as positive control.

### Statistical Analysis

Statistical significance of differences was calculated using Student’s *t* test or least significant difference (LSD) using DPS software (v. 3.11).

## Results

### Gene Isolation and Sequence Analysis

In our previous reports, ten *DkERF* genes (*DkERF1*-*DkERF10*) were isolated, among which DkERF9 and DkERF10 functioned as transcriptional activators of *ADH* and *PDC* promoters [12], [13]. However, additional mRNAs for *ERF* related homologs were discovered that increased in amount during anoxia/deastringency treatment, using RNA-seq. Twelve novel *ERF* genes, designated as *DkERF11* to *DkERF22* (KJ170911-KJ170922), were isolated from ‘Mopan’ persimmon using RNA-seq and RACE.

**Figure 1:**
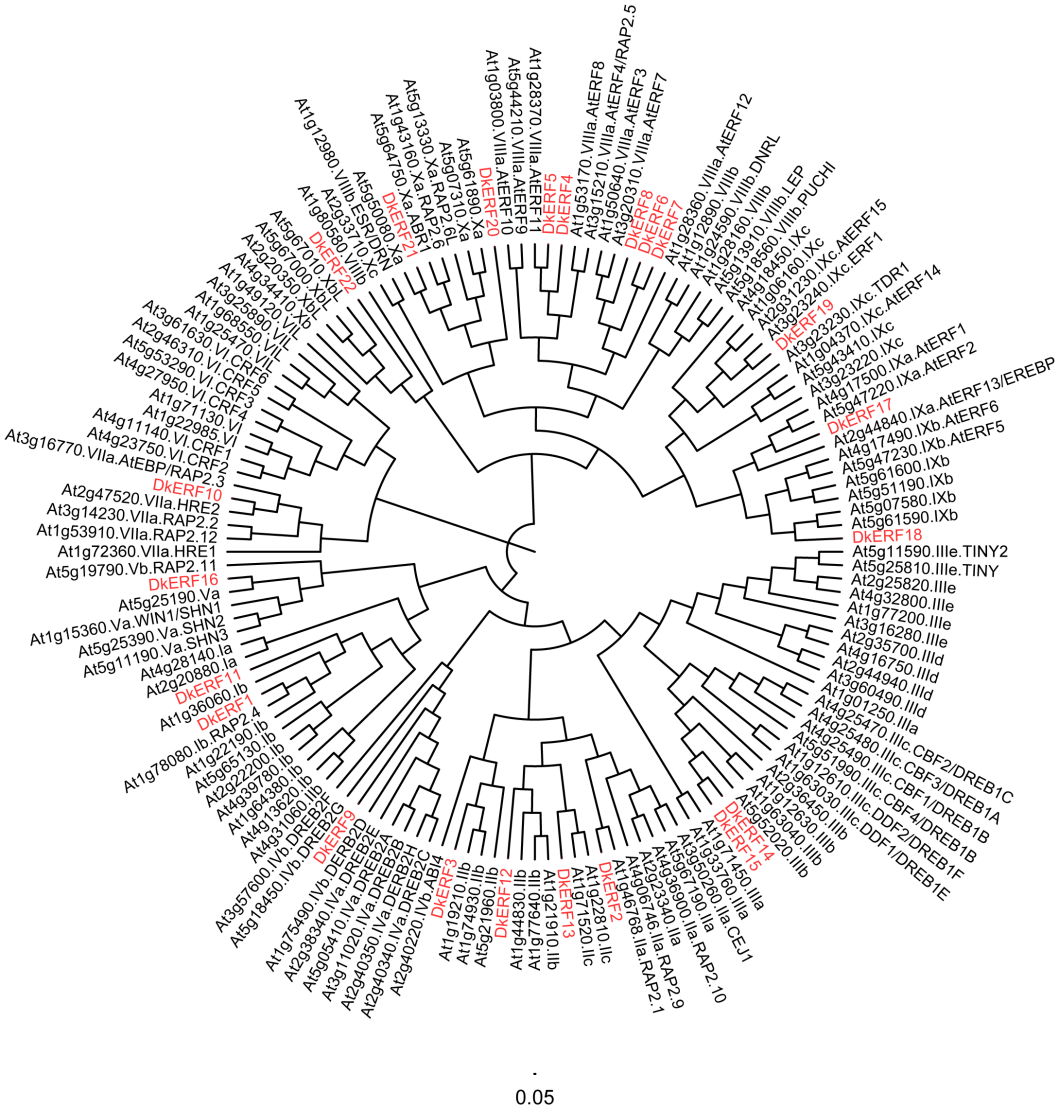
Phylogenetic tree of ethylene response factors. Persimmon *DkERFs* are highlighted in red. The amino acid sequences of the Arabidopsis *ERF* family were obtained from The Arabidopsis Information Resource. The phylogenetic tree was constructed with figtree (version 3.1).

Phylogenetic analysis of the deduced amino acid sequences showed that the 12 *ERF* genes were clustered into seven subfamilies, *DkERF11* was very close to *DkERF1* in subfamily I, *DkERF12* and *DkERF13* belong to subfamily II, *DkERF14* and *DkERF15* belong to subfamily III, *DkERF16* belongs to subfamily V, *DkERF17* -*19* and *DkERF20-22* were clustered in subfamilies IX and X, respectively (Fig. 1).

### Expression Analysis in Relation to Deastringency in ‘Mopan’ Persimmon

**Figure 2:**
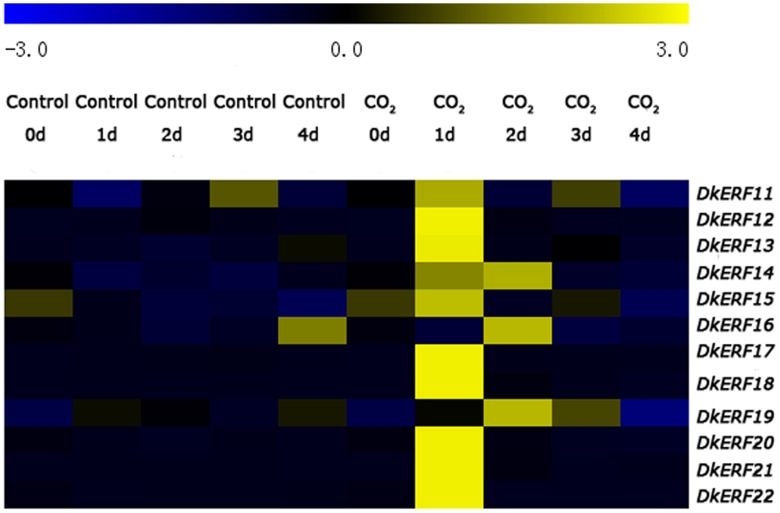
*DkERF* expression patterns in response to CO_2_ (95%, 1 d) treatment in Mopan’ persimmon fruit at 20°C. Relative mRNA abundance was evaluated by real-time PCR. The heatmap indicates the average mRNA abundance from three biological repeats and was constructed by MeV4.8.1.

Since the twelve novel *ERF* genes were indicated as being induced by anoxia/deastringency treatment, realtime PCR was used to confirm the changes in transcript abundance of the *ERF* genes. The results indicated that *ERF11*-*ERF22* transcripts were induced by CO_2_ treatment, whereas they remained constant in control fruit (Fig. 2, Figure S1). Most of the *ERF* genes transcripts reached a peak at 1 d; while three *ERF* genes (*DkERF14*, *DkERF16* and *DkERF19*) were expressed most abundantly at 2 d.

### Expression Analysis in Relation to Deastringency in ‘Gong Cheng-shui Shi’ Fruit

**Figure 3:**
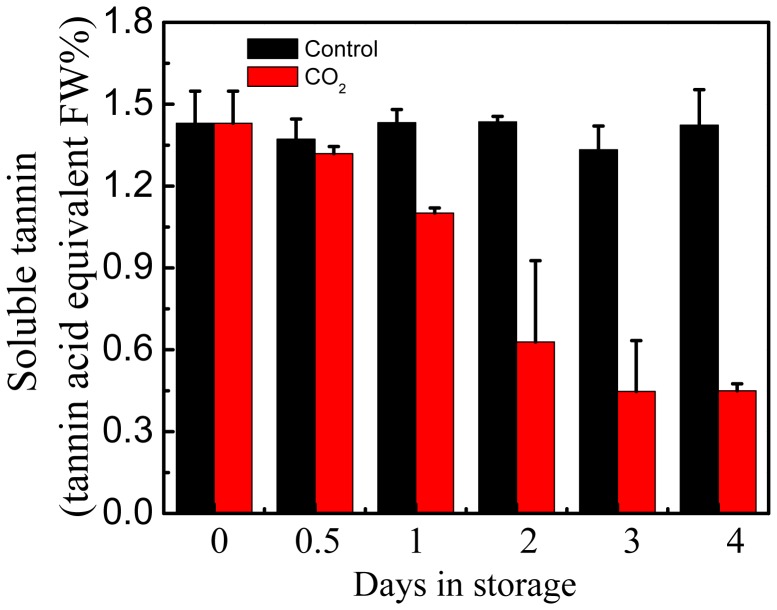
Effects of CO_2_ treatment (95%, 1 day) on soluble tannins in ‘Gong cheng-shui shi’ fruit at 20°C. Black columns and red columns represent the control and the CO_2_ treatment respectively. Error bars represent ±SE from three replicates.

A further experiment was performed with ‘Gong cheng-shui shi’ persimmon, which also respond to deastringency treatment, to confirm the association between *DkERF* genes and deastringency, using CO_2_ (95%), as with ‘Mopan’. The results indicated that CO_2_ treatment accelerated the decrease of soluble tannins content in the flesh of ‘Gong cheng-shui shi’ fruit, as soluble tannins content was much lower in CO_2_-treated fruit (1.101 µg/g at 1 d and 0.450 µg/g at 4 d) than the control fruit (1.432 µg/g at 1 d and 1.423 µg/g at 4 d) (Fig. 3). Fruit softening was also promoted by CO_2_ treatment, as fruit softened from an initial value of 47.375 N to 28.944 N after CO_2_ treatment compared to 46.641 N in control fruit after 4 days in storage (Figure S2).

**Figure 4:**
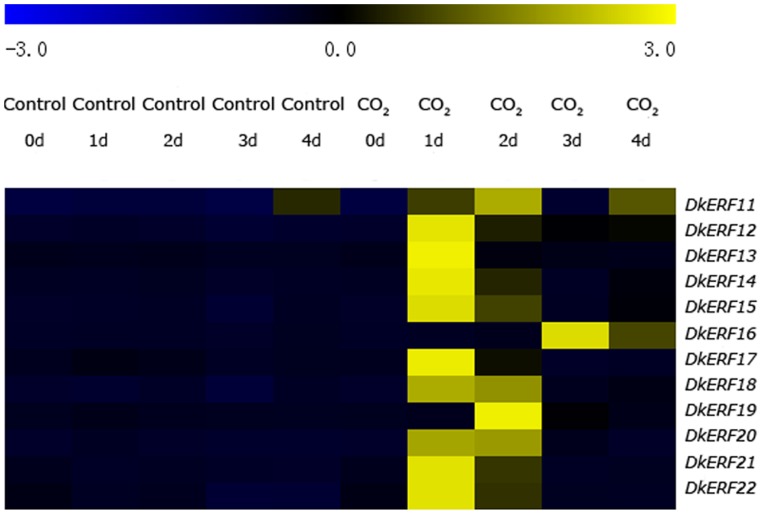
*DkERF* expression patterns in response to CO_2_ (95%, 1 d) treatment in ‘Gong cheng-shui shi’ fruit at 20°C. Relative mRNA abundance was evaluated by real-time PCR. The heatmap indicates the average mRNA abundance from three biological repeats and was constructed by MeV4.8.1.

Concomitantly with the deastringency process (decreasing of soluble tannins), the twelve *DkERF* genes all exhibited an increase in expression. Most of the *DkERF* genes rapidly responded to CO_2_ treatment at 1 d, while transcripts of *DkERF11* and *DkERF19* exhibited delayed accumulation at 2 d, and *DkERF16* peaked at 3 d (Fig. 4, Figure S3). Coordinate changes in *DkERF* genes, *DkADH* and *DkPDC* genes were also induced by CO_2_ treatment (Figure S4). Taking the results from ‘Mopan’ and ‘Gong cheng-shui shi’ fruit together, the gene expression studies showed that increased expression of *DkERF* genes was closely associated with persimmon fruit deastringency.

### Interaction of DkERF and Promoters of Deastringency-related Target Genes

**Figure 5:**
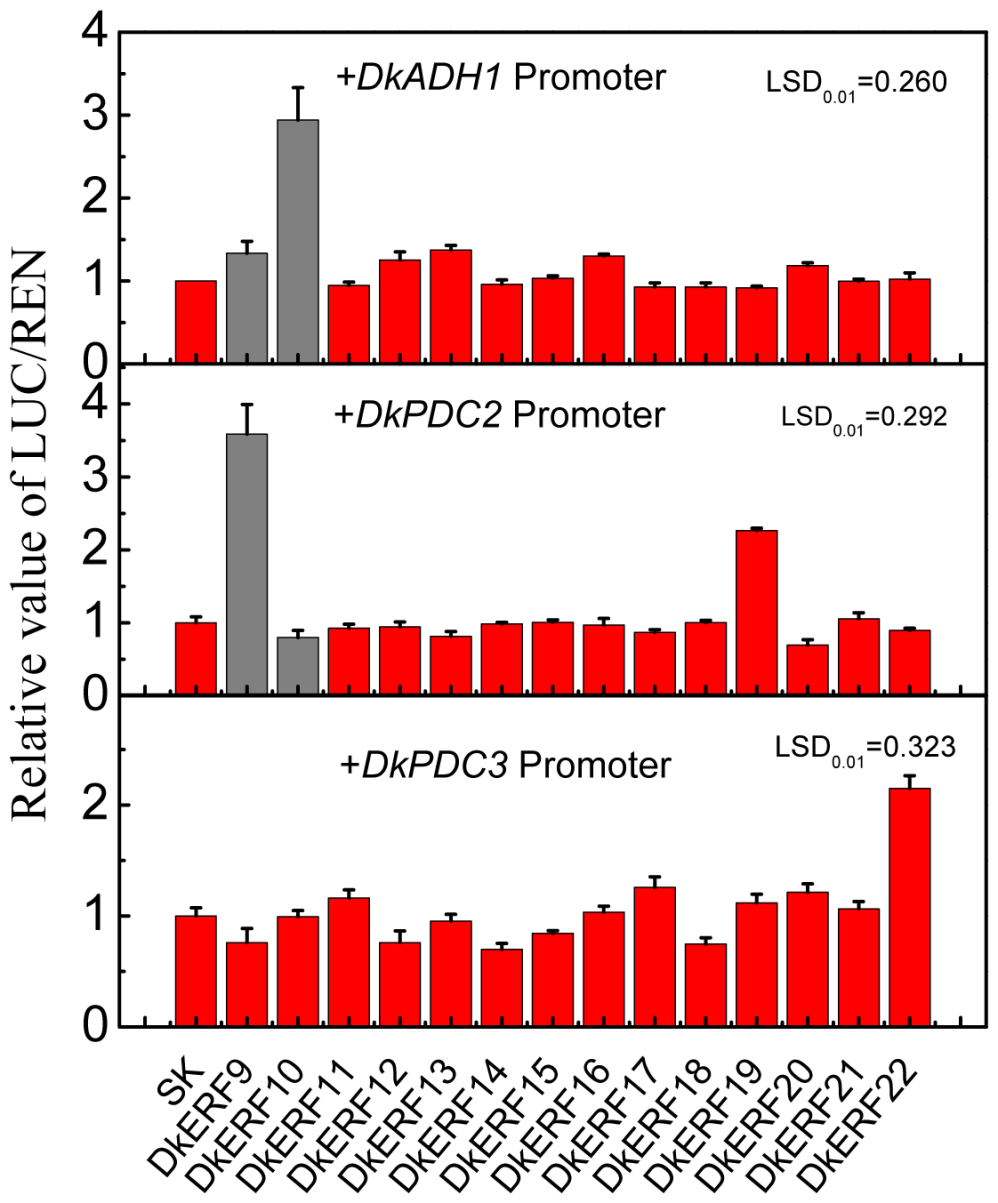
*In vivo* interaction of *DkERF* with the promoters of *DkADH1*, *DkPDC2* and *DkPDC3*. The ratio of LUC/REN of the empty vector (SK) plus promoter was used as calibrator (set as 1). The data in grey columns are derived from Min *et al.*, (2012) for comparison. Error bars indicate±SEs from five biological replicates.

**Figure 6:**
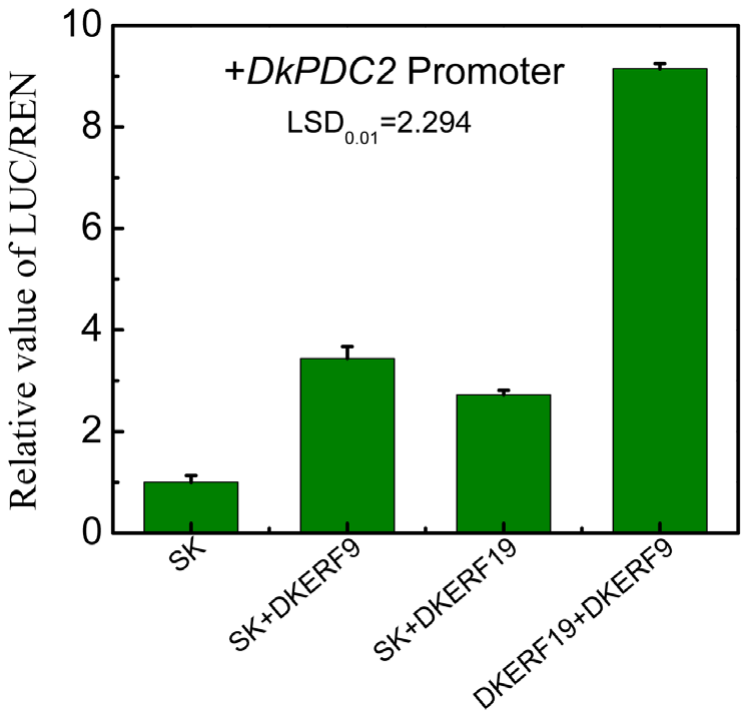
Synergistic trans-activation effect of combination of *DkERF* genes on *DkPDC2* promoter.

**Figure 7:**
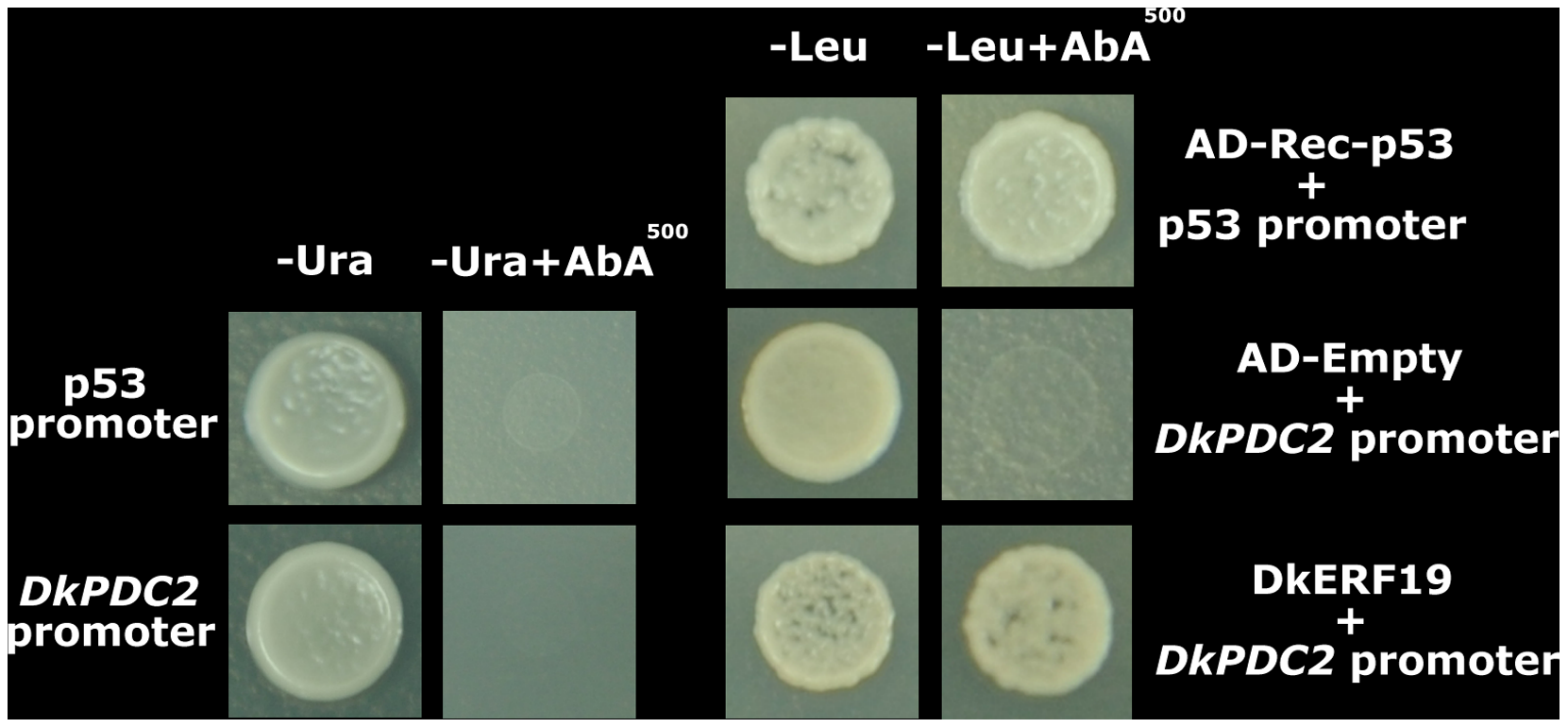
Yeast one-hybrid analysis of *DkERF19* binding to promoter of *DkPDC2*. Auto-activation of promoters were tested on SD medium lacking Ura in presence of aureobasidin A. Interaction was determined on SD medium lacking Leu in presence of aureobasidin A.

On the basis of the initial expression correlation studies, a more direct experiment was performed to test the transcriptional regulatory roles of DkERF on deastringency-related target genes. Firstly, *in vivo* interactions were examined with a dual luciferase assay in *Nicotiana benthamiana* leaves. The previously identified DkERF9 and DkERF10 could activate promoters of deastringency-related genes (Fig. 5). From the novel twelve DkERF, DkERF19 and DkERF22 acted as activators of *DkPDC2* and *DkPDC3*, respectively. No significant interactions were observed between DkERF and *DkADH1*, except for that with the previously characterized DkERF10 (Fig. 5). Moreover, combination of the two activators, DkERF9 and DkERF19, produced a higher activation (LUC/REN = 9.150) than the single transcription factor (LUC/REN values for DkERF9 and DkERF19 are 3.437 and 2.726, respectively) (Fig. 6). Using yeast one-hybrid system, interaction of DkERF19 and DkPDC2 promoter was further confirmed (Fig. 7).

**Figure 8:**
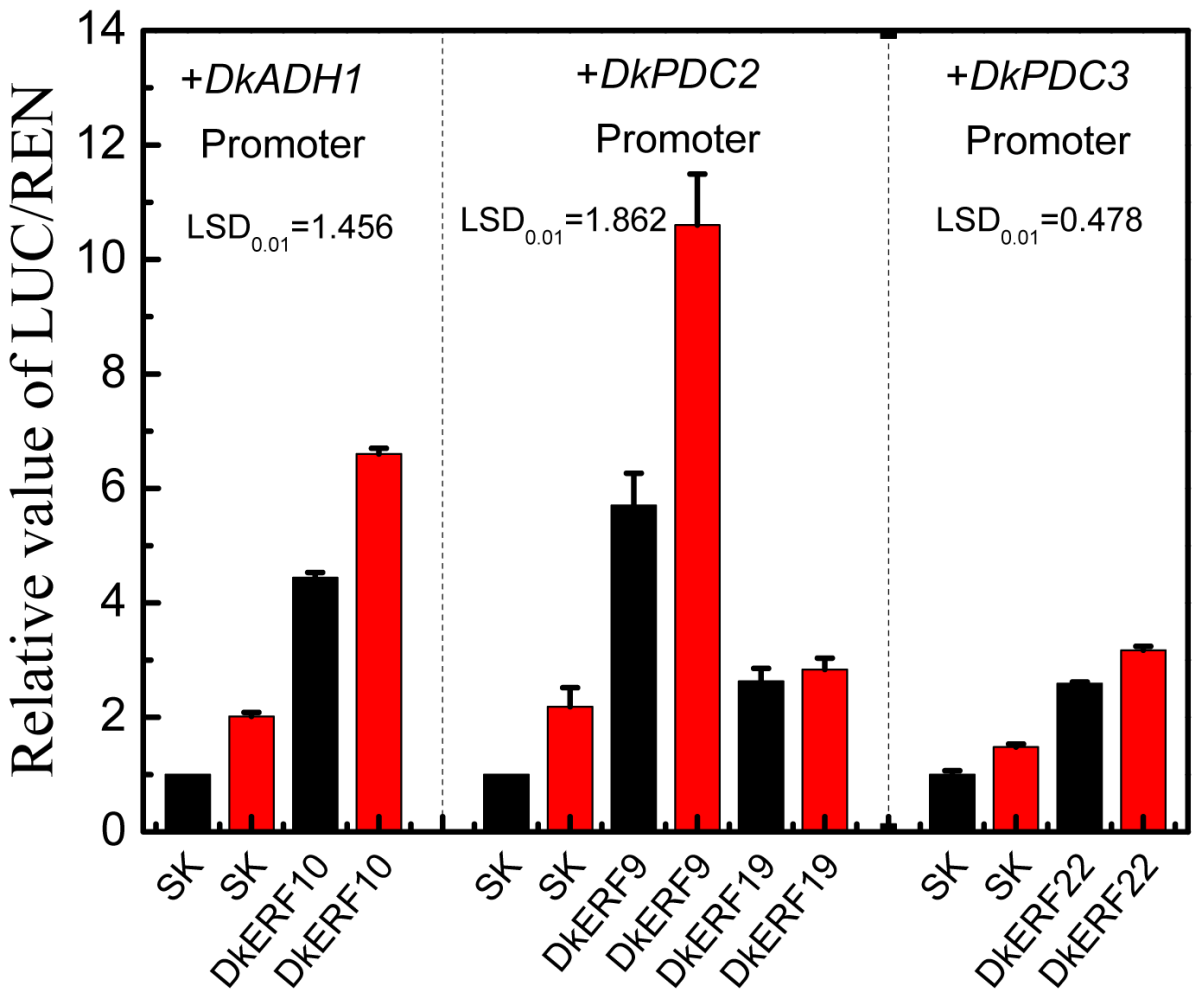
High concentration CO_2_ (95%) affects in vivo interaction of *DkERF* and the *DkADH1* promoter. Tobacco plants, 2% CO_2_ for 12 h. Black and red columns represent control (air) and CO_2_ treatment, respectively. The ratio of LUC/REN of the empty vector (SK) plus promoter was used as calibrator (set as 1). Error bars indicate±S.E.s from five biological replicates.

A further experiment was conducted in order to test the trans-activation activities under the anoxic environment used for deastringency treatment. The results indicated that transcription from the promoters of *DkADH1*, *DkPDC2* and *DkPDC3* were inducible with 95% CO_2_ treatment (supplemented with 4% N_2_ and 1% O_2_). With present of DkERF genes, the relative LUC/REN values were also enhanced with 95% CO_2_ treatment, except for the combination of DkERF19 - *DkPDC2* promoter and DkERF22 - *DkPDC3* promoter (Fig. 8).

## Discussion

Ethylene response factors, the downstream components in the ethylene signal transduction pathway, are encoded by a plant-specific transcription factor gene family [32]. Numerous *ERF* genes have been characterized and shown to be key regulators in plant defense/response to abiotic and biotic stresses, including the newly identified hypoxia-responsive *ERF* [22]. So far, at least five *ERF* genes, including *HRE1*, *HRE2*, *RAP2.2*, *RAP2.3* and *RAP2.12*, have been implicated as key control elements in Arabidopsis tolerance to anoxia, by transcriptional regulation of *ADH* and *PDC* genes [23], [26]. Generally, hypoxia is an undesirable growth environment for most plants, although, on the contrary, an hypoxia/low oxygen environment is one of the most effective treatments to improved persimmon fruit quality for human consumption, because it leads to production of acetaldehyde, which is responsible for removal of the astringency taste caused by soluble tannins [9], [13], [33]. Thus, investigating the roles of hypoxia-responsive *ERF* genes in persimmon fruit should contribute both to the functional analysis of the *ERF* family and also to fruit quality improvement.

Our previous results indicated that two hypoxia-responsive ERF genes, DkERF9 and DkERF10, interacted with promoters of the deastringency-related genes *DkPDC2* and *DkADH1* [12]. However, the results from persimmon showed some differences compared to the Arabidopsis results, where the hypoxia-responsive *ERF* genes were mainly clustered within the subfamily VII [34], while in persimmon *DkERF9* and *DkERF10* they belong to subfamily IV and VII [12]. Thus, we proposed that the role of *ERF* genes in persimmon astringency removal might be more complicated than in the Arabidopsis hypoxia response. Thus, transcriptome analysis by RNA-seq was chosen for the present research, due to the lack of genome information available. Twelve novel *ERF* genes (*DkERF11-22*) were characterized as being responsive to anoxia/deastringency (95% CO_2_) treatment. These *DkERF* genes were widely distributed throughout the *ERF* family, including subfamily I, II, III, VII, IX and X. Moreover, the transcriptional responses of these *DkERF* genes to the CO_2_ treatment were conserved in two different astringent cultivars, cv. ‘Mopan’ and cv. ‘Gong cheng-shuishi’, and the expression patterns were similar to the five hypoxia-responsive *ERF* genes in Arabidopsis [23]. These results, once again, indicated that *ERF* genes involved in persimmon fruit deastringency show some similarities to those in the *Arabidopsis* hypoxia response, but in persimmon the four *DkERF* genes, *DkERF9/10/19/22*, were distributed into subfamilies IV, VII, VIII and IX (Fig. 1), while the hypoxia responsive *ERF* genes were mainly clustered into subfamily VII.

In persimmon, DkERF19 and DkERF22, activated promoters of *DkPDC2* and *DkPDC3* (Fig. 5). Taken together with our previous results, three anoxia-responsive deastringency genes have their own specific regulators, DkERF10 for the *DkADH1* promoter, DkERF9 and DkERF19 for the *DkPDC2* promoter, and DkERF22 for the *DkPDC3* promoter. In Arabidopsis, hypoxia responsive *ERF* positively regulate the *ADH* and *PDC* genes [23], but the effect of anoxia environment on the trans-activation of ERF on the promoters of target genes were not reported. Here, we found that the transient activations of *DkERF* genes on the target promoters were substantially enhanced by deastringency treatment (95% CO_2_). Due to the slight changes were observed from DkERF19-*DkPDC2* promoter and DkERF22-*DkPDC3* promoter, thus the enhancement of 95% CO_2_ on ERF-promoter may not only by turning on the target promoters. However, the mechanisms of thus increasing transient activations need further investigations.

One of the most interesting observations from the present research is the additive effect of DkERF9 and DkERF19 on the promoter of *DkPDC2*. In plants, there are examples of transcription factor complexes, where two or even more transcription factors interact with each other generating stronger activation phenomenon, such as MYB-bHLH-WD40 in anthocyanin biosynthesis [35], [36]. Some new transcription factors interaction have also been reported, such as HD2 and ERF1 in longan fruit [19]. However, The mechanisms of additive effect of DkERF9 and DkERF19 need further research in order to test this possibility.

In conclusion, on the basis of our previous studies and transcriptome analysis, there are at least 18 *DkERF* genes (*DkERF1*, *4*, *5*, *6*, *9–22*) responsive to hypoxia/deastringency treatment (95% CO_2_). Taken together our previous report and the present results, only four DkERF, DkERF9/10/19/22, were characterized as activators for specific target genes. Based on the present findings, we propose that *DkERF* genes involved in persimmon deastringency may partially mimic the similar functions of hypoxia-responsive *ERF* genes in Arabidopsis. The five key *HRE* genes in Arabidopsis, however, belong to subfamily VII, while four of the persimmon *DkERF* genes belong to subfamily IV, VII, IX and X, respectively. These results provided a more comprehensive overview of functions of *DkERF* in persimmon deastringency removal at the transcriptome level, and also provided some hints to isolate more hypoxia-responsive *ERF* genes from other plants.

## Supporting Information

Figure S1 **Expression of** ***DkERF*** **genes in response to CO_2_ treatment in ‘Mopan’ persimmon.** Supplemental to Fig. 2 in manuscript.
(TIF)

Figure S2 **Effects of high concentration of CO_2_ (95%, red open circles, 1 day) treatment on firmness in ‘Gong cheng-shui shi’ fruit at 20°C.** Error bars represent ±SE from ten replicates.
(TIF)

Figure S3 **Expression of** ***DkERF*** **genes in response to CO_2_ treatment in ‘Gongcheng-shuishi’ persimmon.** Supplemental to Fig. 5 in manuscript.
(TIF)

Figure S4 ***DkADH*** **and** ***DkPDC*** **Expression patterns in response to CO_2_ treatment in ‘Gong cheng-shui shi’ persimmon fruit at 20°C.** Relative mRNA abundance was evaluated by real-time PCR from three biological repeats by Mev.4.8.1 soft.
(TIF)

Table S1 **The sequences of primers used for RACE.**
(PDF)

Table S2 **The sequences of primers used for Real-time PCR.**
(PDF)

Table S3 **The sequences of primers used for full-length amplification.**
(PDF)

Table S4 **The sequences of primers used for yeast one-hybrid analysis.**
(PDF)
