# Supplementary figures and images for: Two Novel Anoxia-Induced Ethylene Response Factors That Interact with Promoters of Deastringency-Related Genes from Persimmon
Source: PLoS One. 2014 May 7;9(5):e97043. doi: 10.1371/journal.pone.0097043 (PMC4013125; doi:10.1371/journal.pone.0097043)

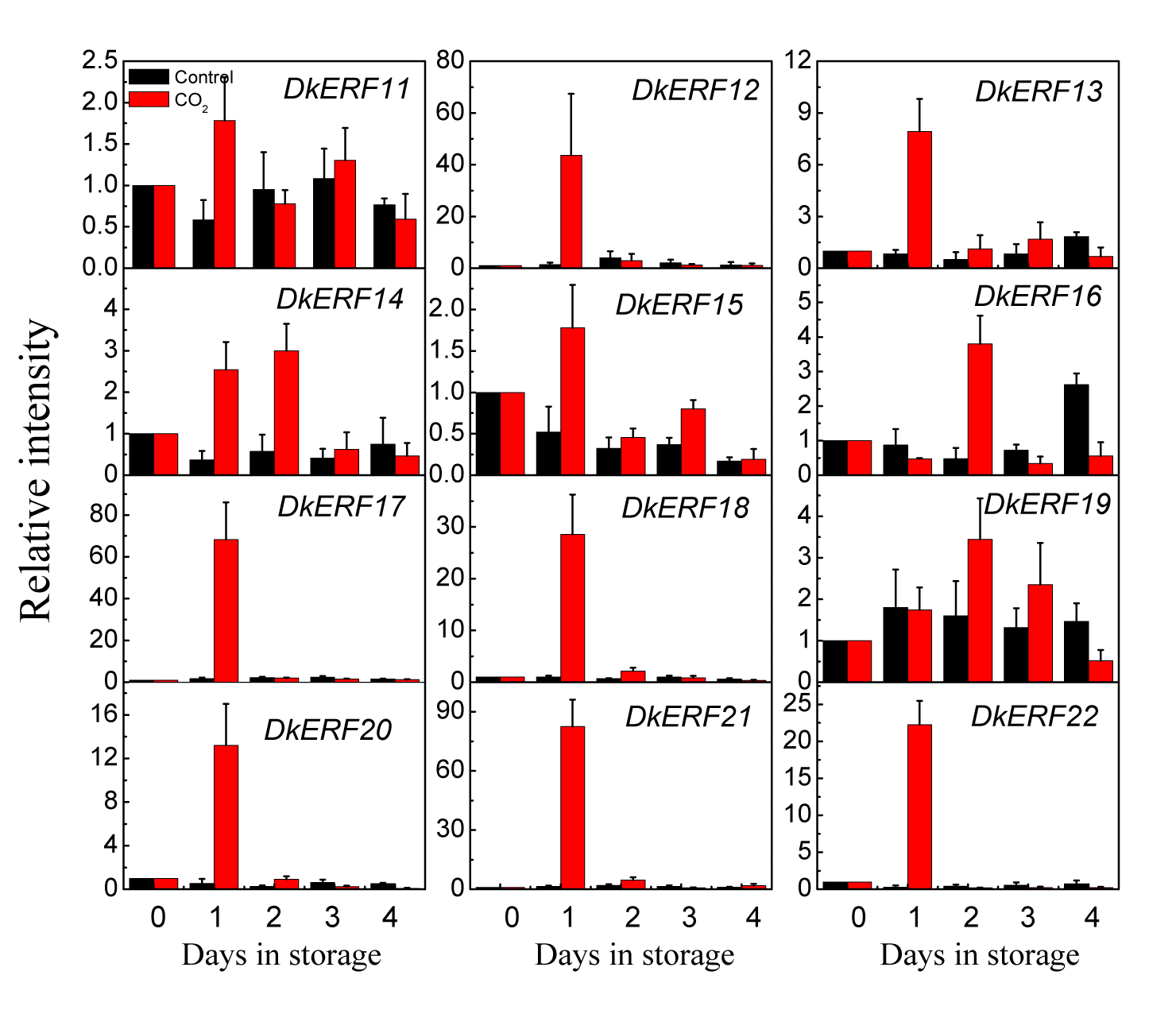

Supplement: Figure S1 — Expression of DkERF genes in response to CO2 treatment in ‘Mopan’ persimmon. Supplemental to Fig. 2 in manuscript. (TIF) [file pone.0097043.s001.tif]

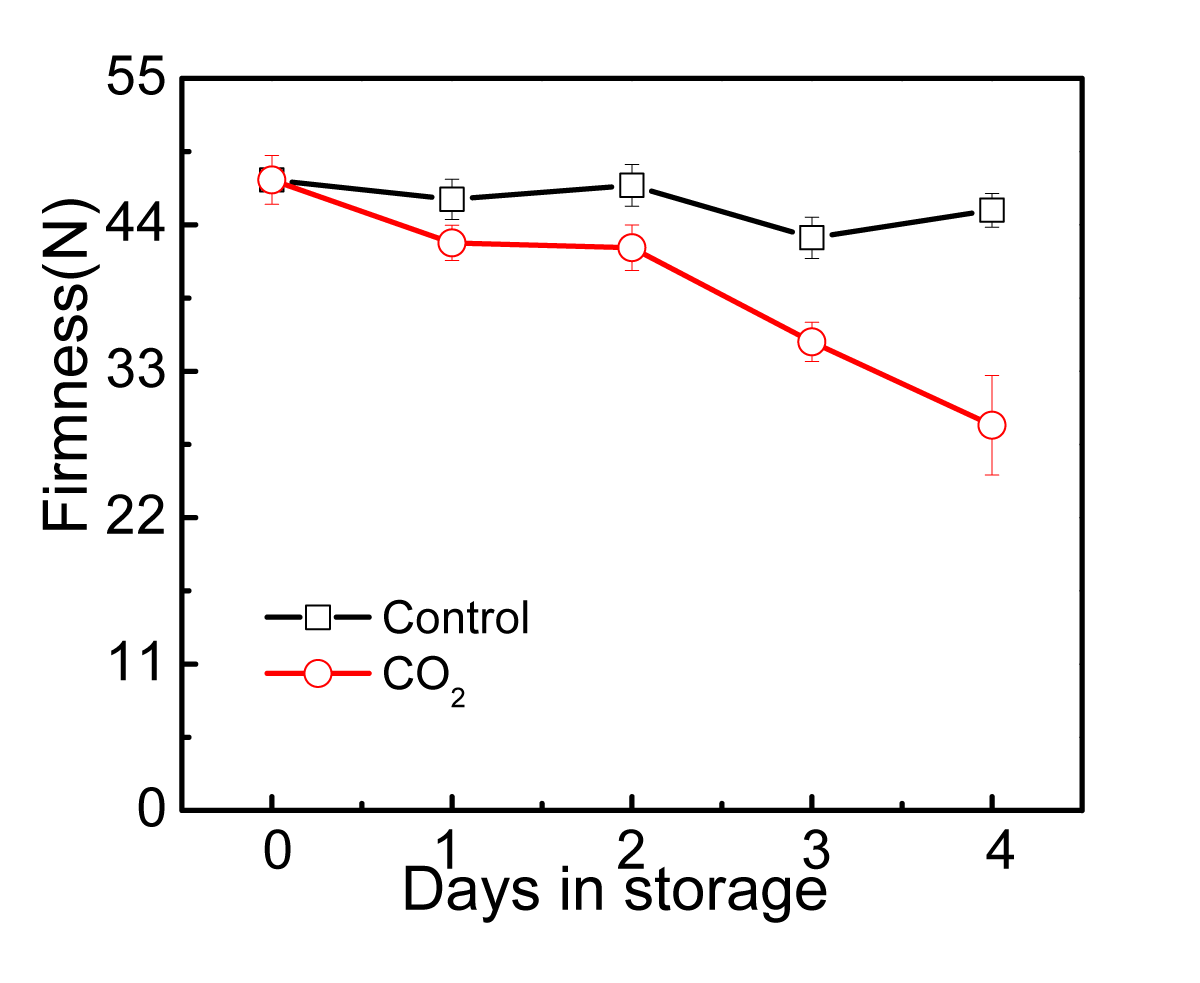

Supplement: Figure S2 — Effects of high concentration of CO2 (95%, red open circles, 1 day) treatment on firmness in ‘Gong cheng-shui shi’ fruit at 20°C. Error bars represent ±SE from ten replicates. (TIF) [file pone.0097043.s002.tif]

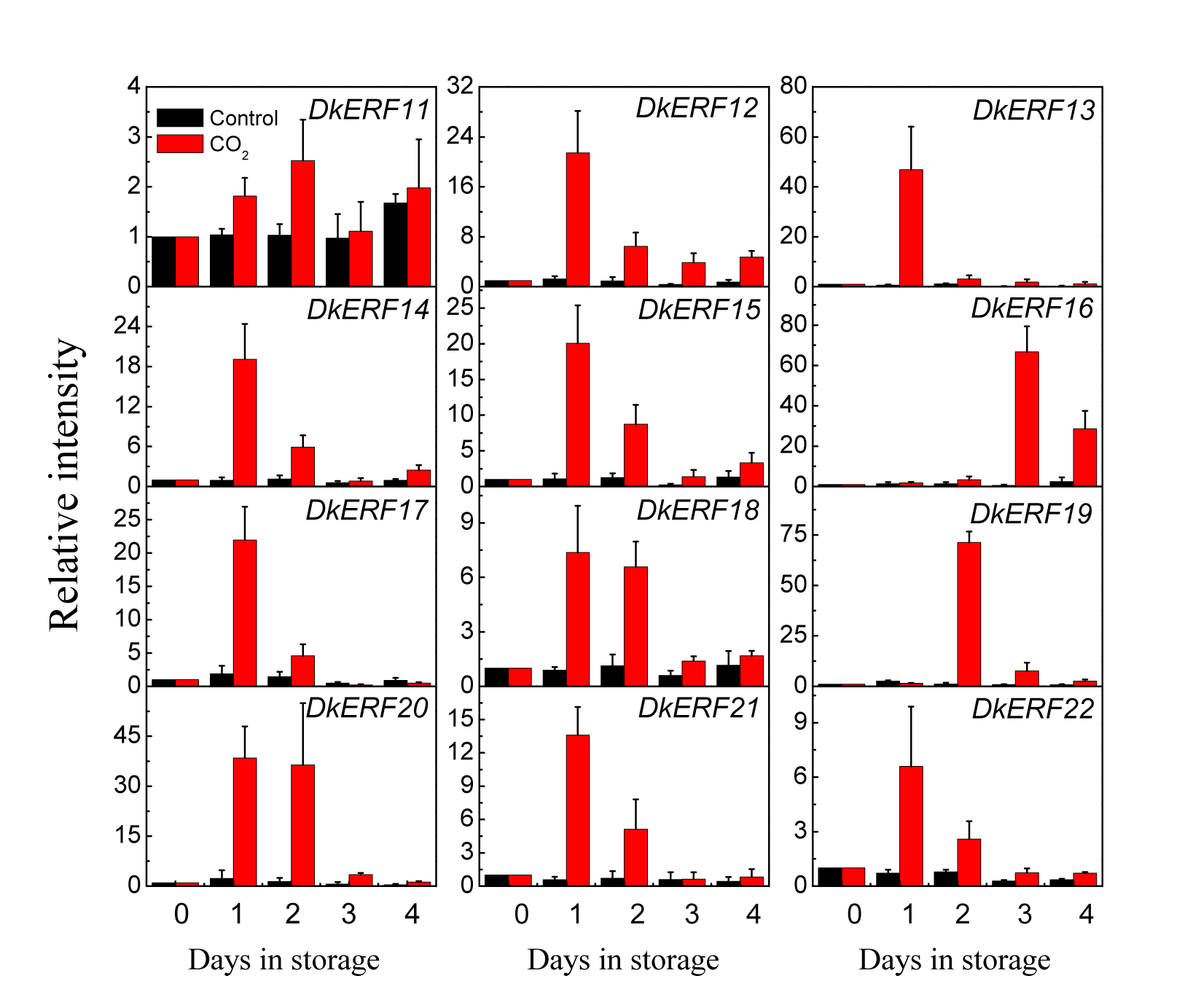

Supplement: Figure S3 — Expression of DkERF genes in response to CO2 treatment in ‘Gongcheng-shuishi’ persimmon. Supplemental to Fig. 5 in manuscript. (TIF) [file pone.0097043.s003.tif]

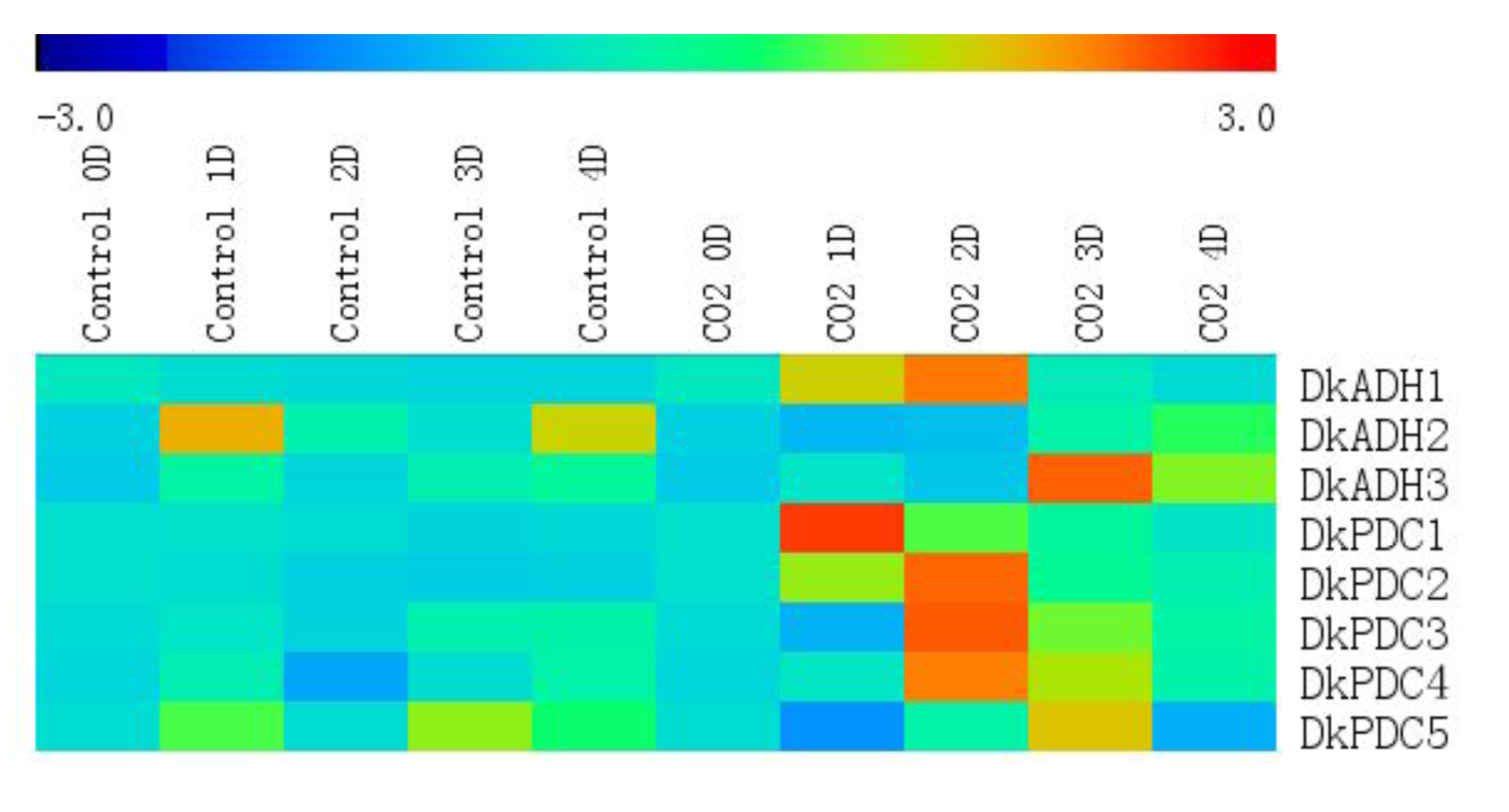

Supplement: Figure S4 — DkADH and DkPDC Expression patterns in response to CO2 treatment in ‘Gong cheng-shui shi’ persimmon fruit at 20°C. Relative mRNA abundance was evaluated by real-time PCR from three biological repeats by Mev.4.8.1 soft. (TIF) [file pone.0097043.s004.tif]
