# Supplementary material for: Two Novel Anoxia-Induced Ethylene Response Factors That Interact with Promoters of Deastringency-Related Genes from Persimmon
Source: PLoS One. 2014 May 7;9(5):e97043. doi: 10.1371/journal.pone.0097043 (PMC4013125; doi:10.1371/journal.pone.0097043)
Supplement: Table S1 — The sequences of primers used for RACE. (PDF) [file pone.0097043.s005.pdf]

Table S1 The sequences of primers used for RACE

	Gene	Primary PCR (5' to 3')	Secondary PCR (5' to 3')
3'RACE	*DkERF11*	TTCCTAGAAGAGTCCCATCCGGTTATG	GTGAGCTCATGGAAGCACTTGAACC
	DkERF12	CATTGATTGATCCTGTCTGGCTGTG	CGACTTCGAGAATGGTGAGGAAGAA
	DkERF13	AACAGCCGATCCGACCAAATCATAC	CCGATCCGACCAAATCATACAGAGG
	DkERF14	ACCGACCAATACCATCAGAATCGAA	GGAAGACAAATTGCCGCATTCTACA
	DkERF15	CGACGGCGATCAAGACTGAAGAAG	CGATCAAGACTGAAGAAGGCGAGAAA
	DkERF16	GAGTTGCTTGATTATGGGTCCATTGAA	GTCCATTGAACTTTGCTCTGTTGTTCC
	DkERF17	ATTCTTACTTACGCCCACAGGCACA	CCCACCCAAATTATCCTATCCCAAA
	DkERF18	TGATGATGATGACCTCGACTGGAAG	CACCTCCTCAGCGACTTCACCTCTA
	DkERF19	GGAGCTTCGTCGGAGAGCAATAA	AGCTTCGTCGGAGAGCAATAATTCG
	DkERF20	CCAGCAGCCATTACTCAGGAGAGAA	GCTGCTCTCAGATTCAAAGGGAACA
	DkERF21	TTACGACGAGGCTGCTCTCAGATTC	ATTCAGAGGAAACAAGGCCAAGCTC
	DkERF22	ACGAAAGCACAAGCGAAACCCTTAC	TTATAACGACGAGCTCCAGCAAAGC
5'RACE	*DkERF13*	TGATTTGGTCGGATCGGCTGTTGTCG	TCGGATACCCACTTCCCCCATTTTCG
	DkERF15	GCCCGTATATCCGCCTGAGACGAAGA	TGAGCCTCCGCCGTCACCTCCTCCTG
	DkERF16	TCATCTGCTCAATGTGGCAATCTTCA	CCTGATGAGATGGAACAACAGAGCAA
	DkERF17	CTCCTTCACTGGCGGCACCATCATTT	TCACTCCCCTGTAGTGCCTCCCCTTC
	DkERF19	TCCGCCAGAACGCCGAACAGCAGCAT	CCTCTTCTTTGTTGCCGCCCCCATTC
	DkERF20	TGGTCGCCGCCTAACCCCTCTATACTG	GCTTCTTGATGAGATGGCAGTGGGTGA
	DkERF22	GCCGACACCATTGCCGACATCTCCAGC	CTTCTCGTCCTCTCTTCCCTCCGCTGC
