# Supplementary material for: Two Novel Anoxia-Induced Ethylene Response Factors That Interact with Promoters of Deastringency-Related Genes from Persimmon
Source: PLoS One. 2014 May 7;9(5):e97043. doi: 10.1371/journal.pone.0097043 (PMC4013125; doi:10.1371/journal.pone.0097043)
Supplement: Table S2 — The sequences of primers used for Real-time PCR. (PDF) [file pone.0097043.s006.pdf]

Table S2 The sequences of primers used for Real-time PCR

Gene	Forward (5' to 3')	Reverse (5' to 3')
DkERF11	TGGGCTTCTCTATAGAACTCTCTT	AAACATGGAGAATGACAAGC
DkERF12	GATGTTTATGAAGGAGAAGCTG	GGCGGGTCTGGGTTTTAT
DkERF13	GATGATCCATAAGAGTCTTGCAG	TGCATGATCAGGAGGTAATATG
DkERF14	AGAGCCTGTGGAGCTATGATTA	ACCTCTCATCACCATTTTGTTC
DkERF15	GAACAAAATGGTGATGAGAGGT	GTTCCCAACTCTTTCGATCTTAG
DkERF16	TCAGGCTATGTGATCTACCTCTC	CACTACTCACAGAGGGGTAAAAAT
DkERF17	CAGAAAGGACAGTTGCCAGT	AAACTAAACAGGGCCCATAGAT
DkERF18	GACGTCGTCTTCCTCGTCTTA	GCCTATTTTAACACTCCACACTG
DkERF19	CCCAGACTCAAAGTATCTGATGA	AAAGACAGGGACATAAGAGAAGG
DkERF20	CAAACGTACTCTCAACAGGTGAT	ACAACTTACTCGAGATACTGCAC
DkERF21	CCTGTAGTTCGCATTTTTGAGT	ACTCGACCAACCTAAAACAAGA
DkERF22	GCAGTAAGAAGCCAAGAATCAT	GAAAAACTGGAGCCGATAGAG
