# Supplementary material for: Two Novel Anoxia-Induced Ethylene Response Factors That Interact with Promoters of Deastringency-Related Genes from Persimmon
Source: PLoS One. 2014 May 7;9(5):e97043. doi: 10.1371/journal.pone.0097043 (PMC4013125; doi:10.1371/journal.pone.0097043)
Supplement: Table S3 — The sequences of primers used for full-length amplification. (PDF) [file pone.0097043.s007.pdf]

Table S3 The sequences of primers used for full-length amplification

Gene	Primary PCR (5' to 3')	Secondary PCR (5' to 3')
DkERF11	TGAGCTCATGGAAGCACTTG	CTATAGAGAAGCCCAATCAATCT
DkERF12	CGACTTCGAGAATGGTGAGGAAGAA	ATCAGCAAAAGCTCCAAAGC
DkERF13	CGAAGATGAGCAGCTCCAGCGAA	TCACAGATAATCTTCCACCGAAA
DkERF14	ATAATGGCTGACCAAATAGACC	GTCTATTTAATTTCTTGGCAA
DkERF15	TATTGGGCTGTCAGCTGCAATGA	GTCTATTAATCATAGCTCCAAAG
DkERF16	CAAACACCTTCTCCCTGCAT	TCACATAGCCTGATGAGATGG
DkERF17	GAGACCGTGTCTATGAGTTTTCC	ATCTAAATAGTCACCACCAATA
DkERF18	ATGATGATGACCTCGACTG	TGCTTTAAGACGAGGAAGACGAC
DkERF19	TGAAAGGAGAAACAAACTCCATG	TAAGGAGTTCATCATCATCAGA
DkERF20	ATGGTCTCAGCTCTAGCTCAAG	ATTAATTGATCCGATAAGTACGA
DkERF21	ATGTGCTGTGAGTTGAAGGTGGCG	TCAAAAATGCGAACTACAGGAAA
DkERF22	ATGGACGGTATTTACGTGAACG	AGAAGGAGAAAATGTTACAAAGC
DkPDC3	GAAATCTTTCTGATCGCAGGAG	CCATGGTCTTACGGACTGGAAAGGAACT
