# Supplementary material for: Two Novel Anoxia-Induced Ethylene Response Factors That Interact with Promoters of Deastringency-Related Genes from Persimmon
Source: PLoS One. 2014 May 7;9(5):e97043. doi: 10.1371/journal.pone.0097043 (PMC4013125; doi:10.1371/journal.pone.0097043)
Supplement: Table S4 — The sequences of primers used for yeast one-hybrid analysis. (PDF) [file pone.0097043.s008.pdf]

Table S4. The sequences of primers used for yeast one-hybrid analysis

Gene	Forward (5' to 3')	Reverse (5' to 3')
DkPDC2 Promoter	GAGCTCTCGACTGATATCGAATCAAATTGAAC	GTCGACCATGGTAATTACTTGAACAGAAGC
DkERF19 Full length	CATATGATGGATTCTTTCTGGATCCACGG	CTCGAGTCATCAGATACTTTGAGTCTGGG
